# Nurses’ self-efficacy and practices relating to weight management of adult patients: a path analysis

**DOI:** 10.1186/1479-5868-10-131

**Published:** 2013-12-04

**Authors:** Da Q Zhu, Ian J Norman, Alison E While

**Affiliations:** 1 Second Military Medical University, School of Nursing, Xiangyin Road, Shanghai, People’s Republic of China; 2 King’s College London, Florence Nightingale School of Nursing and Midwifery, James Clerk Maxwell Building, Waterloo Road, London, UK

**Keywords:** Nurses, Self-efficacy, Weight management practices, Model testing

## Abstract

**Background:**

Health professionals play a key role in the prevention and treatment of excess weight and obesity, but many have expressed a lack of confidence in their ability to manage obese patients with their delivery of weight-management care remaining limited. The specific mechanism underlying inadequate practices in professional weight management remains unclear. The primary purpose of this study was to examine a self-efficacy theory-based model in understanding Registered Nurses’ (RNs) professional performance relating to weight management.

**Methods:**

A self-report questionnaire was developed based upon the hypothesized model and administered to a convenience sample of 588 RNs. Data were collected regarding socio-demographic variables, psychosocial variables (attitudes towards obese people, professional role identity, teamwork beliefs, perceived skills, perceived barriers and self-efficacy) and professional weight management practices. Structural equation modeling was conducted to identify correlations between the above variables and to test the goodness of fit of the proposed model.

**Results:**

The survey response rate was 71.4% (*n* = 420). The respondents reported a moderate level of weight management practices. Self-efficacy directly and positively predicted the weight management practices of the RNs (*β* = 0.36, *p* < 0.01), and fully or partially mediated the relationships between perceived skills, perceived barriers, professional role identity and teamwork beliefs and weight management practices. The final model constructed in this study demonstrated a good fit to the data [χ^2^ (14) =13.90, *p* = 0.46; GFI = 0.99; AGFI = 0.98; NNFI = 1.00; CFI = 1.00; RMSEA = 0.00; AIC = 57.90], accounting for 38.4% and 43.2% of the variance in weight management practices and self-efficacy, respectively.

**Conclusions:**

Self-efficacy theory appears to be useful in understanding the weight management practices of RNs. Interventions targeting the enhancement of self-efficacy may be effective in promoting RNs’ professional performance in managing overweight and obese patients.

## Background

Health professionals play a key role in managing obesity which is a global public health priority [1]. Although most health professionals hold favorable attitudes concerning their role in the prevention and treatment of obesity, many have expressed a lack of confidence in their ability to manage obese patients, and their actual professional weight management practices fall below the recommendations set out in the evidence-based guidelines of several countries, for example, health professionals should use every opportunity to help patients with changes to their lifestyles (dietary advice and exercise) [2-4]. This is evident from studies of patients [5-7], physicians [8-12] and nurses [13,14]. Patient surveys have suggested that less than a half of obese patients were advised by their physicians to lose weight [5-7] with a minority of physicians reporting confidence in helping obese patients lose weight [8], half performing waist measurement [12], and less than half always providing specific guidance on diet, physical activity or weight control for their adult patients [11]. These findings are consistent with those for nurses with few routinely using the body mass index (BMI) to make clinical judgments of weight status or obesity [13,14]. Moreover, longitudinal studies suggest that professional weight management practices among health professionals have declined. Data from the US Behavioral Risk Factor Surveillance System indicated that, despite the 1998 National Institutes of Health guidelines and increases in morbid obesity, the proportion of obese people who reported being counseled by a health professional declined from 42.3% in 1994 to 40.3% in 2000 [5]. Given the increasing prevalence of overweight and obese people globally, it is important to understand the variables which influence health professionals’ weight management practices.

### Variables related to professional weight management practices among health professionals

A small number of studies have examined the factors associated with health professionals’ engagement with weight management within their work. Variables examined include: socio-demographic characteristics, such as gender [9], age [9,15], BMI [16,17], specialties [9,15] and previous training [15,18], and psychosocial characteristics, such as negative attitudes towards obese patients [18,19], perceived barriers [20], perceived skills [21] and self-efficacy [22,23]. However, little theory-based research has been directed towards exploring the interrelationships among the above factors and professional weight management practices of health professionals. For instance, we are aware of only two studies that have employed social cognitive theories to predict nurses’ behaviors related to obesity management [22,23]. Hoppe et al. [22] drew upon an integrated framework derived from self-regulation and planned behavior theories to examine the UK practice nurses' frequency of raising the issue of weight loss with their patients. This early study revealed that self-efficacy was the strongest predictor of nurses’ raising weight loss issues with overweight patients, and perceived barriers to raising the issue were negatively related to self-efficacy. Collectively, the explained variance in the behavior (raising weight loss issue with all overweight patients) was 46%. Boyer [23] drew upon a modified version of the Health Belief Model to examine the nurses’ implementation of weight management approaches and advice strategies. This study also found that self-efficacy related to obesity treatment was positively associated with the consistent performance of weight management care and provision of weight management advice. His proposed model only accounted for 11% of the variance in self-efficacy determination.

Although the two studies provide valuable information regarding nurses’ professional weight management practices, further research is needed for the following reasons: (1) it is unclear whether Hoppe et al.’s findings continue to reflect UK nurses’ attitudes and practices, especially since the launch of the 2006 UK clinical guideline on weight management to improve the care provided by health professionals to adults and children with obesity [4], and the 2008 national education program to help people maintain a healthy weight [24]; (2) Boyer’s research did not examine several important psychosocial variables which may account for the small portion of variance in the professional weight management behaviors explained by the proposed model; (3) neither study examined the influence of the group’s contextual factors on nursing practice, such as teamwork beliefs, which might be an important predictor of nurses’ professional practice since a multidisciplinary team approach to weight management is recommended [4].

### Conceptual framework

**Figure 1:**
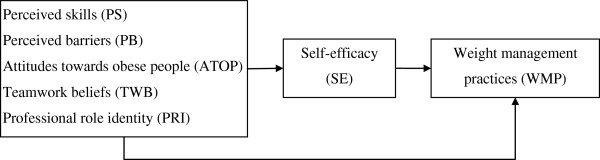
Hypothesized model of the relationships among the study variables.

In light of the limitations of the previous studies we hypothesized that a new theoretical perspective incorporating self-efficacy, attitudes towards obese people, perceived barriers, perceived skills, professional role identity and teamwork beliefs may provide a fuller understanding of the professional weight management practices of health professionals. The conceptual model underpinning this study draws upon Bandura’s Self-Efficacy Theory [25] and previous empirical findings which hypothesize a direct pathway from distal psychosocial factors to professional weight management practices and an indirect pathway via self-efficacy (Figure 1).

According to Bandura’s [25] theory, self-efficacy (SE), defined as belief in one’s capabilities to organize and execute the courses of action required to manage prospective situations, is the most central and pervasive influence on the choices that people make and the amount of effort that they apply to a particular task. In this study, self-efficacy referred to a nurse’s confidence to perform professional weight management behaviors for overweight and obese patients. Self-efficacy has been consistently identified as a critical predictor of clinical performance, such as the administration of opioids for pain relief [26], providing diabetes care [27], counseling performance [28] and obesity management [22,23]. A systematic review of 78 studies which investigated health professionals’ behaviors from a social cognition perspective found that beliefs about capabilities was one of the most frequent predictors of health professionals’ behaviors [29]. Thus, we hypothesized that self-efficacy would directly and positively predict professional weight management practices.

There is also considerable evidence that self-efficacy is a mediator in the context of work performance including professional nursing practices [27,30]. For example, a study of a random sample of 500 North American nurses, which examined the relationship between structural empowerment, nursing leadership and self-efficacy and its effect on nursing practice, found that self-efficacy partially mediated the relationship between structural empowerment and professional practice behaviors [30]. Similarly, studies of nurses’ providing diabetes care and reducing catheter occlusion have shown that nurses’ self-efficacy is related to knowledge and relevant clinical behavior [27], which suggests a possible mediation effect of self-efficacy on the relationship between knowledge and behavior. Although no such correlation has been described in studies of professional weight management, this study hypothesized that self-efficacy would play a mediating role in predicting or explaining professional weight management practices.

The construct of perceived skills (PS) was defined in this study as a nurse’s perception of skills needed to treat overweight and obese patients. Our assumption was that health professionals who perceived themselves to have adequate skills would have reasonable confidence in their ability to perform clinical activities and were more likely to use these with their patients. Links between perceived skills, self-efficacy and professional behaviors have been established [23] and have been supported by intervention studies showing that skill-based training or education can improve health professionals’ self-efficacy and facilitate their clinical performance [31]. Thus, we hypothesized that nurses’ perceived skills related to weight management would not only directly and positively influence their professional weight management behaviors, but would also indirectly influence the target behavior through self-efficacy.

In this study perceived barriers (PB) referred to a nurse’s belief about the material and psychological costs of practicing weight management care. Some studies have shown that health professionals’ perceived barriers can impede professional weight management practices. Shay et al. [20] found that primary care providers were unlikely to raise weight or weight control strategies with their patients once they perceived barriers to actions, such as too little time, not enough training and lack of financial incentive. Similarly, a sample of 620 primary care physicians reported that they would spend more time on weight management issues if their time was reimbursed [8]. A negative association between perceived barriers and self-efficacy was also found for practice nurses’ counseling for weight loss [22]. Therefore, we hypothesized that nurses’ perceived barriers to obesity management would have a direct and negative effect on their weight management practices, as well as an indirect effect through self-efficacy.

The construct of attitudes towards obese people (ATOP) was defined as nurses’ perceptions of personal characteristics of obese individuals, including positive (e.g., industrious, pleasant and graceful) and negative components (e.g., lazy, unpleasant and awkward). Negative weight-based attitudes towards obese patients have been reported among physicians, nurses, psychologists, dieticians, and medical students [19,32]. It is not known how these negative attitudes (e.g. weight-based stigma, prejudice, and discrimination) among health professionals affect their confidence and behaviors in providing treatment to overweight and obese patients. However, some studies have indicated that obese patients are reluctant to seek medical care, and may be more likely to delay important preventative healthcare because of their experiences of weight-related discrimination in healthcare settings [33]. This study assumed that attitudes towards obese patients would be directly associated with professional weight management practices and indirectly associated when mediated by self-efficacy.

Teamwork beliefs (TWB) and professional role identity (PRI) were introduced for the first time in this study as the context of weight management practices. The former was defined as a nurse’s beliefs about working collaboratively with colleagues to deliver weight management care, and the latter as a nurse’s perceptions of role pressure to perform or not to perform weight management care. Although the relationships between TWB and PRI and weight management care have not been previously evaluated, a few studies have suggested the influence of role identity and teamwork on other healthcare behaviors, such as administration of opioids for pain relief [26] and provision of professional labor support to parturient women [34]. We therefore hypothesized that positive teamwork beliefs and professional role identity would be directly associated with more frequent professional weight management practices and higher levels of self-efficacy.

Throughout this study, the term weight management practices (WMP) encompasses the recommended clinical practice activities of assessment, advice, support, and referral specific to obese and overweight adults set out in the UK and Chinese clinical guidelines [4,35,36]. For example, BMI should be used to classify overweight or obese adults.

## Methods

### Aims

Overall, this study aimed to test a proposed model of the interrelationships among six psychosocial factors and weight management practices of RNs using a path analysis based on structural equation modeling. We hypothesized that: (1) perceived skills, perceived barriers, attitudes towards obese people, professional role identity and teamwork beliefs would directly influence weight management practices; and (2) self-efficacy would be the central factor, directly influencing weight management practices and mediating the relationships between distal psychosocial factors and weight management practices.

### Study design and participants

A convenience sample of 588 British RNs attending courses at a large London university were surveyed using a self-completion questionnaire. Of the 420 (71.4%) nurses who responded, 21 provided unusable questionnaires due to the lack of key information, resulting in a total of 399 valid responses. Since the survey was anonymous it was not possible to compare the demographic profiles of respondents and non-respondents.

Ethical approval for the study was obtained from the university research ethics committee. All respondents gave informed consent prior to their inclusion in the study and did not receive any financial compensation for their participation.

### Measures

A questionnaire was developed to measure potential factors underpinning weight management practices based upon a review of the literature. Subsequently, a pilot study with 14 RNs with expertise in obesity management confirmed that the questionnaire items were unambiguous and appropriate and that the questionnaire could be completed within 30 minutes. The final questionnaire comprised five scales to examine six psychosocial factors (attitudes towards obese people, professional role identity, teamwork beliefs, perceived skills, perceived barriers, self-efficacy) and weight management practices. Socio-demographic data were also collected (e.g., age, gender, ethnicity and clinical specialties).

Table 1 provides a summary of the scales included together with their psychometric properties. Psychometric analyses were conducted for each scale used in this study. First, an item analysis was computed to determine the critical ratio (CR). The items having statistically significant CR values (*t* > 1.96, *P* < 0.05) were retained in the scale. Second, exploratory factor analysis using principle components analysis was conducted to assess the underlying structure of each scale. The number of factors selected was based on the scree test and a minimum eigenvalue of 1.00. A factor loading of at least 0.40 was selected as the criterion for determining which items contributed to a given factor. Internal consistency (Cronbach’s α) was calculated for the whole instrument and its potential factors. Finally, test–retest reliability over a two week period was tested by calculating the intra-class correlation coefficient (ICC) for each Likert scale by using study data from 34 targeted respondents. In summary, our psychometric testing indicated that the study scales had acceptable internal consistency, interpretable and adequate factor structure and good stability over two weeks, and were therefore considered reliable and valid measures of the variables in the study.

**Table 1:** Psychometric analyses of the scales used in the present study

**Scales**	**Conceptual factors**	**Item numbers on factor**	**% variance explained**	**Factor loadings**	**Alpha in the testing sample**	**ICC**	**Scoring**^**a **^**(*M*** **± SD)**	**Actual range**	**Possible range**
Attitudes Towards Obese Persons	Attitudes towards obese people (ATOP)	20	-	-	0.81	0.87	68.08 ± 16.27	23-116	0-120
Attitudes Towards Weight Management	Self-efficacy (SE)	8	30.5	0.46-0.80	0.82	0.80	33.82 ± 6.93	14-48	8-48
	Professional role identity (PRI)	4	13.1	0.64-0.80	0.80	0.60	19.79 ± 3.42	4-24	4-24
	Teamwork beliefs (TWB)	5	8.4	0.59-0.78	0.73	0.69	25.97 ± 3.52	11-30	5-30
Perceived Barriers	Perceived barriers (PB)	8	44.4	0.52-0.78	0.81	0.78	34.97 ± 6.72	8-48	8-48
Perceived Skills	Perceived skills (PS)	7	50.9	0.52-0.82	0.83	0.83	13.76 ± 3.44	7-21	7-21
Weight Management Practices	Weight management practices (WMP)	8	52.6	0.58-0.85	0.86	0.77	22.21 ± 7.94	8-40	8-40

^a^All the scores were computed by summing all items except for ATOP scores.

#### Attitudes towards obese persons (ATOP)

This scale was derived from Allison et al.’s [37] 20 item, 6-point Likert scale (-3 = strongly disagree, 3 = strongly agree) to measure attitudes toward obese people. As recommended [37], items indicative of negative attitudes towards obese people were multiplied by -1 and then 60 was added to the sum of the responses to all items. Thus, a total score may range from 0 to 120. Higher scores indicate more positive attitudes. In this study, the Cronbach’s α was 0.81 close to that reported by Allison et al.

#### Attitudes towards weight management (ATWM)

The ATWM scale is a 25-item 6-point Likert scale (1 = strongly disagree, 6 = strongly agree). Most items were adapted from the Improving the Nutrition and the Care of the Overweight Patient Survey questionnaire [38] and others were added by the authors. After item analysis and factor analysis, 17 items were retained, reflecting a logical three-factor structure: self-efficacy (SE), professional role identity (PRI) and teamwork beliefs (TWB) in delivering weight management care. The SE sub-scale comprised eight items relating to nurses’ perceptions of their confidence or capability to deliver professional weight management practices (e.g. ‘I can determine patients’ BMI and assess whether they are overweight or obese’). The PRI sub-scale comprised by four items which explored nurses’ perceptions of “themselves” in relation to their professional role in managing obese and overweight people (e.g. ‘Nurses should take more responsibility for health promotion’). The five-item TWB sub-scale focused on team values and measured the belief that the team involving GPs, dietitians and nurses can perform effectively (e.g. ‘Specially trained nurses are valuable in providing weight reduction diets for patients’). The values of Cronbach’s α for the whole scale were 0.85 and for three sub-scales 0.82, 0.80 and 0.73, respectively.

#### Perceived barriers (PB)

This nine-item scale rated on a 6-point Likert scale (1 = strongly disagree, 6 = strongly agree) was developed for this study based on our literature review [16,17] to measure commonly reported barriers to weight management practice (e.g., lack of time, lack of compensation and complex patients). Factor analysis revealed eight items loading on a single factor, and the items were summed to determine the PB score. The Cronbach’s α for the scale was 0.81.

#### Perceived skills (PS)

This was developed for the study to measure seven skills recommended for the prevention and treatment of overweight or obesity patients (e.g. weight assessment skills, counseling skills and referral skills ) [4,35], with item responses ranging from 1 = low level to 3 = high level. Factor analysis indicated a single factor, and the items were summed to produce the PS score. The Cronbach’s α for the scale was 0.83.

#### Weight management practices (WMP)

WMP was measured using a 8-item Likert scale developed for the study drawing on Brown et al.’s [13] questionnaire and behavioral goals for the management of obesity and overweight identified in recent UK and Chinese guidelines [4,35,36] including the identification and provision of brief interventions and also the referral to other services (e.g., BMI/waist circumference assessment, counseling for diet/exercise and emotional support). The respondents indicated the percentage of their patients who received each of eight clinical activities, using a 5-point scale (1 = 0%, 5 ≥ 75%). The factor analysis revealed a single factor accounting for 52.6% of the variance. The scores of all items were summed to produce the WMP score, which measured the frequency with which patients received each clinical activity delivered by nurses. The Cronbach’s α for the scale was 0.86.

### Statistical analyses

Data were analyzed using SPSS V.17.0 and LISREL 8.7. Scale scores were used as indicators for each construct in the proposed model.

Missing values, normality and outliers were checked prior to analysis. Of the 399 questionnaires, 43 cases (10.8%) had missing values. Given that data were missing at random, missing data were estimated using all other variables in the dataset as predictors for the Expectation Maximization algorithm, which has been proven more robust than mean substitution and regression imputation [39]. All the study variables were normally distributed with Skewness and Kurtosis below 1.96, therefore, parameter estimations for the path model were generated via maximum likelihood estimation.

Pearson’s correlations were used to explore the relationships between the study variables, which provided insight into how the various variables may affect one another and helped to identify an initial model. For each estimated model, we controlled for the influence of demographics by treating these variables as covariates. Path analysis was conducted to test the fit of the model. Of those models which showed sufficient compatibility, the model with the lowest Akaike information criterion (AIC) value was adopted as the final model.

Two analyses were tested for mediation. Generally, the causal steps approach is recommended, in which a series of requirements must be met to suggest that a mediation effect has occurred: (1) the initial predictor is associated with the outcome and the proposed mediator, (2) the mediator is associated with the endpoint of interest outcome; and finally, (3) the initial predictor loses (or substantially diminishes) its effect on the endpoint once the mediator is added as a second predictor in the structural equation model. The Sobel test was then used to evaluate the significance of mediation effect which examined the null hypothesis of no difference between the total effect and the direct effect. An effect is partially mediated when the indirect effect is smaller and of the same sign as the total effect.

## Results

### Socio-demographic characteristics

The characteristics of the nurse sample are summarized in Table 2. Few demographic variables were statistically significantly related to the study variables (not shown). The nurses with previous training or working in community reported higher WMP scores than those without previous training (27.05 vs. 21.24, *p* < 0.01) or worked in hospital (26.40 vs. 21.51, *p* < 0.01). These two demographic variables were added to the model as covariates to test if the relationships among study variables were independent of demographic factors.

**Table 2:** Sample characteristics

***Characteristics***^**a**^	***n *** **(%)**
Gender	
Female	354 (88.7)
Ethnicity	
White	220 (56.4)
Asian and mixed Asian	69 (17.7)
Black and mixed Black	101 (25.9)
Education level	
Diploma of higher education or less	148 (40.0)
Bachelor degree	163 (44.1)
Master or higher	59 (15.9)
Specialties	
Surgery	73 (20.2)
Medical	160 (44.2)
Others	129 (35.6)
Work place	
Hospital	320 (88.6)
Community	41 (11.4)
Previous training in weight management (yes)	64 (16.0)
	*n* (*M* ± SD)
Age	399 (36.25 ± 8.85)
Years in practice	399 (10.12 ± 7.93)
BMI	399 (24.97 ± 4.78)

^a^Total numbers may not add to 399 because not all respondents completed all items.

### Beliefs, attitudes and practices related to weight management

Table 1 lists study conceptual variables, the mean, standard deviation and the range for scale scores. The mean ATOP score of 68.08, slightly higher than the mid-point, suggested neutral attitudes towards obese people. The mean scores of ATWM factors were 33.82 (SD = 6.93) for SE, 19.79 (SD = 3.42) for PRI and 25.97 (SD = 3.52) for TWB, respectively, indicating a moderate level of self-efficacy, and strong role identity and teamwork beliefs with respect to weight management. A high mean score (*M* = 34.97, SD = 6.72) for the PB scale indicated high levels of perceived barriers to weight management. Overall the nurses rated their skills (*M* = 13.76, SD = 3.44) and weight management practices (*M* = 22.21, SD = 7.94) as moderate.

### Bivariate analysis

Table 3 shows the Pearson correlation coefficient matrix of the measured variables in the model. Most variables were significantly correlated with each other. As expected, SE was positively associated with PRI (*r* = 0.45, *p* < 0.01), TWB (*r* = 0.30, *p* < 0.01), and PS (*r* = 0.57, *p* < 0.01), but negatively associated with PB (*r* = -0.12, *p* < 0.05). WMP was positively associated with PRI (*r* = 0.28, *p* < 0.01), TWB (*r* = 0.11, *p* < 0.05), PS (*r* = 0.52, *p* < 0.01) and SE (*r* = 0.56, *p* < 0.01). However, a significant association was not found between ATOP and SE or WMP, nor between PB and WMP (*r* = -0.08, *p* > 0.05).

**Table 3:** **Pearson correlation coefficients matrix of study variables (*n*** **= 399)**

	**ATOP**	**PRI**	**TWB**	**PB**	**PS**	**SE**	**WMP**
Attitudes towards obese people (ATOP)	1						
Professional role identity (PRI)	-.06	1					
Teamwork attitudes (TWB)	-.15^**^	.45^**^	1				
Perceived barriers (PB)	-.12^*^	-.01	.20^**^	1			
Perceived skills (PS)	.03	.29^**^	.13^**^	-.12^*^	1		
Self-efficacy (SE)	-.01	.45^**^	.30^**^	-.12^*^	.57^**^	1	
Weight management practices (WMP)	.03	.28^**^	.11^*^	-.08	.52^**^	.56^**^	1

p^*^ < 0.05; p^**^ < 0.01.

### Path analyses

**Figure 2:**
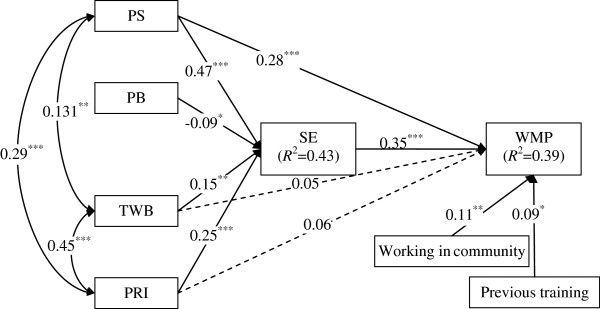
**The initial model of the relationships among the study variables*.****Note*: Arrows represent links, with significant paths shown as solid lines and non-significant paths shown as broken lines. PS: perceived skills; PB: perceived barriers; TWB: teamwork beliefs; PRI: professional role identity; SE: self-efficacy; WMP: weight management practices. p^*^ < 0.05; p^**^ < 0.01; p^***^ < 0.001.

Drawing on the hypothesized model and significant correlations from the bivariate analysis, the following variables were used to construct an initial model: PRI, TWB, PB, PS, SE and WMP (Figure 2). This model revealed a good fit to the data: Chi-square χ^2^ (12) =11.81 (*p* = 0.46), GFI = 0.99, AGFI = 0.98, NNFI = 1.00, CFI = 1.00, RMSEA = 0.00, AIC = 59.81. In the initial model, two direct paths from PRI to WMP (*β* = 0.06, *p* > 0.05) and from TWB to WMP (*β* = 0.05, *p* > 0.05) became non-significant, indicating a fully mediating effect of SE. A partially mediating effect of SE on the relationship between PS and WMP was observed and further confirmed by the Sobel test (*z* = 5.85, *p* < 0.01). Examination of our initial model indicated that adjustment could be made to improve the match between the data and the model. We eliminated two non-significant paths respectively to find the most parsimonious model. All significant paths were entered into a final model to examine the relative importance of the direct and indirect effects on nurses’ behaviors related to weight management care. Table 4 summarizes the estimated models’ goodness of fit indices.

**Table 4:** Goodness of fit indices for the models tested

**Model**	**χ**^**2**^**/*df***	***p***	**GFI**	**AGFI**	**NNFI**	**CFI**	**RMSEA**	**AIC**
Initial model (model 1)	11.81/12	0.46	0.99	0.98	1.00	1.00	.00	59.81
Model 2: remove path from TWB to WMP	13.27/13	0.43	0.99	0.98	1.00	1.00	.00	59.27
Final model (model 3): remove path from PRI to WMP	13.90/14	0.46	0.99	0.98	1.00	1.00	.00	57.90
*Accepted reference value*	<2.0	≥0.05	≥0.95	≥0.90	≥0.95	≥0.95	≤0.05	—

TWB: teamwork beliefs; PRI: professional role identity; WMP: weight management practices.

**Figure 3:**
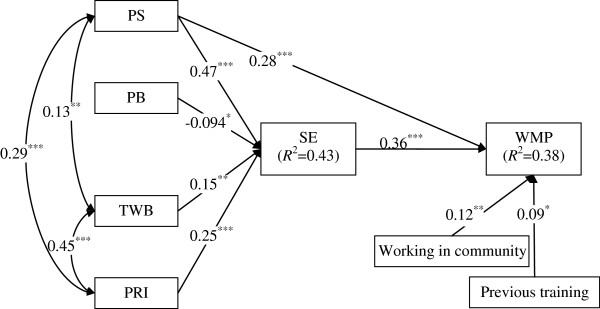
**The final model of the relationships among the study variables.***Note*: PS: perceived skills; PB: perceived barriers; TWB: teamwork beliefs; PRI: professional role identity; SE: self-efficacy; WMP: weight management practices. p^*^ < 0.05; p^**^ < 0.01; p^***^ < 0.001.

The final model with significant pathways and correlations are illustrated in Figure 3. Sufficient model compatibility was indicated with the following values: Chi-square χ^2^ (14) =13.90 (*p* = 0.46), GFI = 0.99, AGFI = 0.98, NNFI = 1.00, CFI = 1.00, RMSEA = 0.00. The AIC value was the lowest in the current model (57.90), indicating the best fit of all the models tested. The final model accounted for 38.4% of the variance in weight management practices and 43.2% of the variance in self-efficacy related to managing excess weight and obesity. As expected, SE was closely associated with WMP (*β* = 0.36, *p* < 0.01), and mediated distal psychosocial factors to WMP: three direct paths from PB, TWB and PRI to WMP were all non-significant, suggesting full mediation of SE; the standardized beta of the direct path from PS to WMP was 0.28 (*p* < 0.01) and the z-value provided by the Sobel test was 6.31 (*p* < 0.01), suggesting partial mediation of SE. However, the data were somewhat inconsistent and no significant direct relationship between distal psychosocial factors and WMP was found, except for that between PS and WMP (*β* = 0.28, *p* < 0.01). Additionally, there was a positive correlation between PS and TWB and PRI, and two covariates (previous training and working in community) contributed significantly to the model.

## Discussion

This study prospectively examined nurses’ weight management practices using self-efficacy theory as a construct framework. The structural equation model constructed in this study largely confirmed our conceptual framework, and extended our understanding of the mechanism through which psychosocial factors affect professional nursing behaviors related to weight management.

### Self-efficacy

As anticipated, self-efficacy was a key factor that directly and positively influenced the weight management practices of the sample RNs. This finding echoes Bandura’s [25] proposition that motivation and performance are determined by how successful people believe they can be, and offers further support to the previous findings of a positive and strong link between health professionals’ performance and their domain-specific self-efficacy [9,22,23]. In addition, the data supported the mediating role of self-efficacy between perceived skills, perceived barriers, teamwork beliefs, professional role identity and weight management practices. This result is similar to those of other studies which found that self-efficacy was a mediator in other healthcare behaviors [27,30] and further highlights self-efficacy as the central psychosocial factor underpinning weight management practices in healthcare settings. Interestingly, we observed relatively high levels of self-efficacy among our sample of nurses compared to samples of previous studies [8,13]. It is possible that health policies and initiatives in the UK designed to improve health professionals’ knowledge and skills related to weight management, such as the Healthy Weight, Healthy Lives program [24], have also improved the nurses’ confidence although this is speculative. The specific reasons for this need further study.

### Perceived skills, training and work place

Perceived skills directly and positively influenced self-efficacy and the weight management practice of the nurses, indicating that those nurses with higher levels of skills were more likely to be confident in their ability to care for overweight and obese patients and more frequently performed weight management than those with lower levels of skills. Our results are consistent with previous studies documenting these relationships between skills, self-efficacy and professional behaviors [23,31,40], and to some extent reflect Bandura’s [41] viewpoint that people's beliefs about their efficacy can be effectively developed through mastery experiences, skills and knowledge.

Working in the community positively associated with weight management practices in this study. This may reflect that the UK has a well-developed system of primary care nursing within which the issue of obesity has been increasingly prioritized over the last two decades [2,4], with nurses as part of primary care teams assessing patient risk and expected to deliver weight and other lifestyle related advice. Thus, nurses working in the community are very likely to provide weight-management interventions for adults, to a greater extent than nurses working in hospitals. This inference still needs to be confirmed in further study.

Also previous training positively predicted weight management practice, which is consistent with a survey of primary care clinicians which found that obesity training during medical school education or residency was strongly related to higher rates of providing diet or exercise counseling to obese patients [18]. These findings suggest that additional training to enhance current practitioners’ weight management skills may facilitate the delivery of obesity-related care. However, less than a fifth of the respondents reported that they had received relevant training, which suggests a need for increased educational opportunities, such as postgraduate education or training for current practitioners to enhance their capabilities related to obesity prevention and treatment, and obesity-related teaching in undergraduate and graduate curricula for future practitioners.

### Professional role identity and teamwork beliefs

It is widely held that collaboration and teamwork impact upon healthcare behaviors (especially complex behaviors) and patient outcomes, and weight management is no exception. The study findings suggest a direct and positive influence of professional role identity/teamwork beliefs on self-efficacy but failed to identify a direct effect on weight management practices. That is to say, the nurses who saw weight management care as part of their role and valued teamwork in weight management practices were more likely to report higher levels of self-efficacy, which might in turn promote their behaviors related to weight management care. Indirect support for our findings are provided by Godin et al. [29] who found that health professionals’ role identity was indirectly associated with medical behaviors via intention and Lee et al. [42] who reported a positive correlation in a sample of 1996 Korean nurses between nursing performance and collective efficacy (reflecting individuals’ perceptions of the team’s capabilities, one dimension of the attitudes toward teamwork).

This is a novel finding and may open a new avenue for possible improvement in weight management care. For example, to deliver better weight management practices, nurses should recognize the importance of teamwork and multidisciplinary working in their management of obesity. Additionally healthcare organizations should measure nurses’ teamwork beliefs and address the development of multidisciplinary working within future training programs.

In addition, a significant association was identified between perceived skills and professional role identity and teamwork beliefs, suggesting that higher levels of skills may enhance nurses’ role identity and help develop positive views of teamwork and vice versa. These potential correlations need to be further tested in the future studies. Finally, our nurse sample overall agreed that nurses were appropriate health personnel to contribute to weight management care and should take responsibility for providing support and care to patients who are obese. They further believed that multidisciplinary cooperation between GPs, physicians, specialized dieticians and clinical psychologists with the involvement of family members could lead to the better health management of obese people.

### Perceived barriers

Perceived barriers had a significant but weak effect on self-efficacy in this study, which is consistent with previous studies reporting that perceived barriers to obesity management of patients are associated with lower levels of motivation and perceived ability to manage overweight and obesity in adults [20]. However, unlike some earlier studies, this study did not find a direct association between perceived barriers and weight management practices. Perhaps, this discrepancy is due to the differences in the study samples. As a whole, our sample reported frequent barriers to weight management in their practice settings which may have masked or weakened more significant associations between perceived barriers, self-efficacy and weight management practices. Additional training to enhance weight management skills and access to clear clinical practice guidelines to facilitate the delivery of obesity-related care, as well as additional incentives to deliver weight management care, may be an effective strategy for influencing nursing weight management practices in view of the effect of PB on WMP via SE.

### Attitudes towards obese people

Previous studies have reported that health professionals hold similar negative attitudes as the general public towards obese people [19,32], which may hinder the development of trusting relationships between patients and health professionals and in turn compromise the effectiveness of health promotion interventions [43]. However, we did not find that the nurses’ attitudes towards obese patients had a significant effect on their self-efficacy and weight management practices. This finding contrasts with a study which reported that clinicians who did not agree that obesity was a disease were less likely to counsel obese patients in a positive context [18], but is consistent with an integrative review of 15 studies between 1990 and 2007 [19] which concluded that, although health staff hold negative attitudes towards obese people, the impact of these attitudes on the delivery of care or treatment decisions is relatively small. In addition, we were encouraged by the finding that our RN sample held neutral to positive attitudes towards obese people; a finding which confirms those of an earlier study of nurses in England [13].

### Strengths and limitations

The current study is unique in two respects. First, to our knowledge, it is the first study of the structural interrelationship between six psychosocial factors (self-efficacy, attitudes towards obese people, perceived barriers, perceived skills, professional role identity and teamwork beliefs) and behaviors relating to obesity management; professional role identity and teamwork beliefs have not been previously evaluated in relation to weight management practices. The statistically significant paths and the well-fitting model provide empirical support for the study hypotheses and offer a direction for future research. Second, no previous studies have analyzed weight management behavior through the application of structural equation modeling. This is an important development because the construction of a structural equation model with high validity using model compatibility as an index enables the estimation of a main explanatory variable from among candidate factors and a calculation of the total effect of the impact of this variable on outcome.

Although this study contributes to the knowledge of nurses’ self-efficacy and professional practice in weight management, several limitations may limit the generalizability of our findings. First, our sample was selected from one university nursing school in London. Although the sample RNs represented different practice areas and clinical settings across London, generalization of the findings to other population of nurses should be cautious. Second, much unexplained variance remains so that ongoing effort is needed to identify additional variables that might increase the explanatory power of the model. Additionally, the use of a self-administered questionnaire presents the possibility of response bias. Further, although the scales used had moderate to good reliability and construct validity, more research is needed to establish the validity and reliability of instruments that measure nurses’ attitudes, beliefs and practices concerning weight management, including those that collect observations of nurses’ clinical practice activities. Finally, the established structural equation model was based on cross-sectional data which limits the ability to draw causal inferences about the relationships between the study variables. For instance, engagement in the target behaviors may influence the psychosocial variables; for example, the delivery of weight management care may influence feelings of self-efficacy, vice versa. Thus, the findings should be interpreted in terms of statistical prediction only, although our results indicate the proposed model is a plausible representation of the relationship between the constructs. Future research should examine the causal relationships by using experimental or longitudinal methodologies.

## Conclusions

To our knowledge this is the first study to use path analysis to systematically examine the structural relationships between various relevant psychosocial factors and weight management practice. The results suggest that self-efficacy theory is a useful framework for understanding the clinical weight management practices of RNs. Self-efficacy was the most proximate determinant of weight management practices and played a mediating role in the relationship between other psychosocial factors and the target behavior. Of the studied variables, perceived skills were most strongly correlated with self-efficacy and weight management practices in this study; this was followed by professional role identity. The study results suggest priorities for training, education and advocacy efforts to improve the professional weight management practices of nurses. Further research is warranted employing different cognitive conceptions (e.g., collective efficacy, intentions to undertake weight management) and using more objective measures of the variables of interest. Finally, the reported frequency of clinical weight management practices was relatively low indicating the need to increase RNs’ awareness of their role in this important area of practice. The study should help inform the development of tailored interventions to improve nurses’ self-efficacy and facilitate their professional practices related to weight and obesity management.

## Competing interests

The authors declare that they have no competing interests.

## Authors' contributions

DQZ collected and analyzed the data, and drafted the manuscript. IJN contributed to the study design and interpretation and revised the manuscript. AEW contributed to the study design and data collection and had primary responsibility for the final content. All authors read and approved the final manuscript.
